# Repurposing ketoconazole as an exosome directed adjunct to sunitinib in treating renal cell carcinoma

**DOI:** 10.1038/s41598-021-89655-w

**Published:** 2021-05-13

**Authors:** Jacob W. Greenberg, Hogyoung Kim, Ahmed A. Moustafa, Amrita Datta, Pedro C. Barata, A. Hamid Boulares, Asim B. Abdel-Mageed, Louis S. Krane

**Affiliations:** 1 Departments of Urology, Tulane University School of Medicine, 1430 Tulane Ave, New Orleans, LA 70112 USA; 2 Department of Pharmacology, Tulane University School of Medicine, New Orleans, LA 70012 USA; 3 Department of Internal Medicine, Section of Hematology/Oncology, Tulane University School of Medicine, New Orleans, LA 70012 USA; 4 College of Nursing and Health, Loyola University New Orleans, New Orleans, LA 70118 USA; 5 The Stanley Scott Cancer Center/Louisiana Cancer Research Center, School of Medicine, Louisiana State University Health Sciences Center, New Orleans, LA USA; 6 Zoology and Entomology Department, Faculty of Science, Helwan University, Cairo, 11790 Egypt

**Keywords:** Cancer therapeutic resistance, Drug development, Targeted therapies, Cancer microenvironment, Cancer therapy, Metastasis, Urological cancer, Renal cancer, Renal cell carcinoma, ESCRT, Membrane trafficking, Exocytosis, Drug safety, Target identification, Renal cancer, Renal cell carcinoma, Cancer, Cancer microenvironment, Cancer therapy, Urological cancer, Drug development, Preclinical research, Translational research, Cancer, Cancer microenvironment, Cancer therapy, Urological cancer, Cancer, Drug discovery, Diseases, Medical research, Oncology, Renal cell carcinoma, Renal cancer, Urology, Urological cancer, Renal cancer, Cell biology, Organelles, Endosomes

## Abstract

Renal Cell Carcinoma (RCC) is the most common form of kidney cancer, with clear cell RCC (ccRCC) representing about 85% of all RCC tumors. There are limited curable treatments available for metastatic ccRCC because this disease is unresponsive to conventional targeted systemic pharmacotherapy. Exosomes (Exo) are small extracellular vesicles (EVs) secreted from cancer cells with marked roles in tumoral signaling and pharmacological resistance. Ketoconazole (KTZ) is an FDA approved anti-fungal medication which has been shown to suppress exosome biogenesis and secretion, yet its role in ccRCC has not been identified. A time-course, dose-dependent analysis revealed that KTZ selectively decreased secreted Exo in tumoral cell lines. Augmented Exo secretion was further evident by decreased expression of Exo biogenesis (Alix and nSMase) and secretion (Rab27a) markers. Interestingly, KTZ-mediated inhibition of Exo biogenesis was coupled with inhibition of ERK1/2 activation. Next, selective inhibitors were employed and showed ERK signaling had a direct role in mediating KTZ’s inhibition of exosomes. In sunitinib resistant 786-O cells lines, the addition of KTZ potentiates the efficacy of sunitinib by causing Exo inhibition, decreased tumor proliferation, and diminished clonogenic ability of RCC cells. Our findings suggest that KTZ should be explored as an adjunct to current RCC therapies.

## Introduction

Rates of presentation for metastatic renal cell carcinoma (RCC) have remained unchanged in the past quarter century. The lack of standard screening tests and often limited symptoms results in high rates of metastatic disease at the time of diagnosis^1^. Cytotoxic chemotherapy, surgical excision, or radiotherapy are incapable of controlling the disease at this stage. This is due to RCC’s lack of sensitivity and the development of resistance over time^2–4^. Thus, there is a need to delineate the mechanisms of disease progression and resistance.

Exosomes (Exo) are extracellular vesicles, 50–150 nm in diameter, released after fusion of multivesicular bodies to the cell membrane. Exosomes can be isolated from biological fluids including urine and serum^5–7^. Exo contain functional mRNAs, non-coding RNAs, proteins, DNA, enzymes, and lipids to transport this cargo to recipient cells. Multiple publications continue to delineate the role Exo play in the regulation of several physiological processes such as tumor growth, angiogenesis, metastasis, drug resistance, and stem cell maintenance^6–10^. Whether exosomes expelled from resistant cancer cells can confer drug resistance to non-resistant cells, is yet to be fully determined. We recently demonstrated that the trafficking of oncogenic factors by prostate cancer derived Exo subvert the tumor microenvironment and prime the oncogenic reprogramming of tumor-tropic PC patient derived adipose stem cells in vivo^11^. Additionally, we found that the lead compounds’ were validated as potent inhibitors and activators of Exo biogenesis and/or secretion. This creates a potential utility of drug-repurposing as novel adjunct therapeutic strategies in advanced cancer^12,13^.

Systemic targeted treatments for clear cell renal cell carcinoma (ccRCC) are often initially effective patients with metastatic disease but complete durable responses are very rare^13^. Mechanisms of development of chemoresistance remain elusive for patients with disease outside the kidney^3^. Manipulation of exosomes is particularly attractive due to their role in initiation, growth, progression, and drug-resistance of cancers involving interactions with the microenvironment by transferring oncogenic proteins and nucleic acids^7,14,15^. Currently, there are no drugs that selectively target pathways involved in exosome biogenesis and secretion by cancer cells and their uptake by recipient cells. Ketoconazole is an FDA approved anti-fungal medication which we have shown to inhibit pathways of exosomal biogenesis and secretion^13^. Based on those preliminary studies, we examined the mechanism and effect of ketoconazole on biogenesis and secretion of exosome in several RCC cell lines along.

## Results

### Characterization and particle concentration of the plasma-derived exosomes for RCC patients

**Figure 1:**
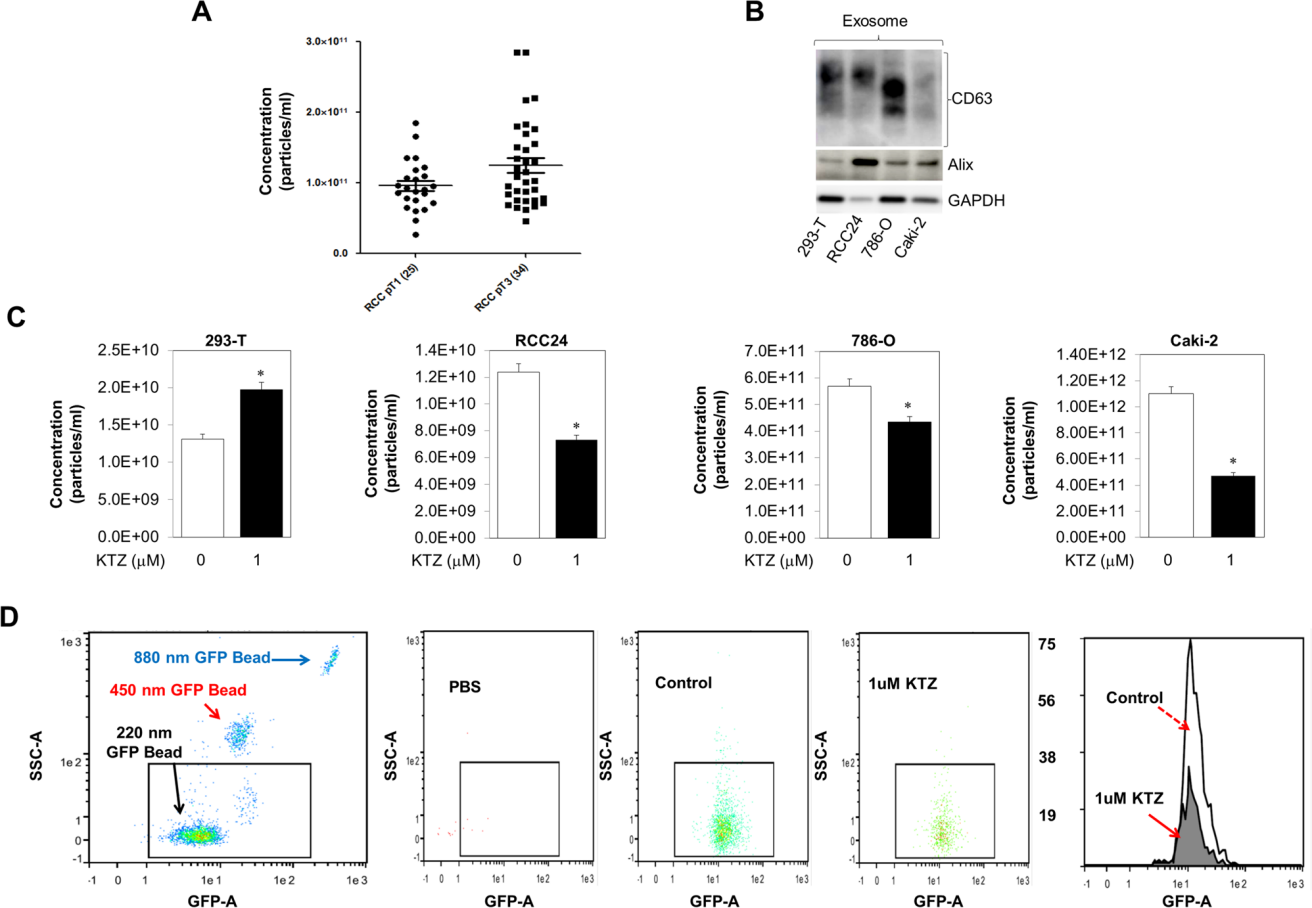
Measurement of exosome secretion by qNano analysis. (**A**) Total number of EVs in the plasma of patients with Kidney cancer and matched controls were measured by a qNano analysis (*n* = 59). (**B**) Expression of exosome CD63, and exosome biogenesis markers Alix in the exosome of 293-T, RCC-24, 786-O, and Caki-2 cells. Exosomes were isolated by UC and filtration (0.45 μm). Full blots are presented in Supplementary Fig. S6 (**C**) qNano-IZON particle quantitative analysis (NP-100 nanopore) depicting a significant decrease in exosome concentrations (50–200 nm size) in the CM of RCC-24, 786-O, and Caki-2 cells treated with KTZ compared to vehicle treated controls, but not normal kidney HEK 293-T cells. *Denotes significance at p < 0.05 compared to controls and was calculated using GraphPad Prism. (**D**) Inhibition of exosome secretion using the MACSQuant Analyzer 10 Flow Cytometer and qNano-IZON: The exosome in the conditioned media were isolated and analyzed by the MACSQuant Analyzer 10 Flow Cytometer as described in the “Materials and methods” section. Treatments were at concentrations of 1 μM ketoconazole compounds. Exosome from DMSO treated 786-O-CD63-GFP is used as GFP control and PBS served as a reference control. *Denotes significance at p < 0.05 compared to controls and was calculated using GraphPad Prism. Data for EVs and exosomes was captured using IZON’s Control Suite software version 3.4.2.48. This software can be found at https://support.izon.com/how-can-i-get-the-latest-software-release.

Exosomes from plasma of 25 patients with pT1a pathological staging (ccRCC renal masses less than 4 cm) and 34 patients with pT3a/b Clear cell renal cell carcinoma (CCRCC) (locally advanced disease) were evaluated using the Tunable Resistive Pulse Sensing (TRPS or qNano) technology. Size ranges and mean sizes of exosome particles were similar in the two groups (with a size range of approximately 50–200 nm, mean diameter ranging from 74–91 nm). The average particle concentration was significantly higher in patients with pT3a/b CCRCC (12.5E10 particles/mL) when compared to patients with pT1a (9.7E10 particles/mL) (p < 0.01), as shown in Fig. 1A. We note RCC patients with aggressive disease have significantly higher levels of circulating exosomes than patients with no evidence of disease.

### KTZ inhibits exosome biogenesis and secretion by RCC cells

MTT cell viability assays were carried out to assess the cytotoxic effects of increasing doses of KTZ (0–10 µM). These physiologically achievable concentrations of KTZ were used to treat RCC-24, 786-O, Caki-2, and HEK 293-T cells (Fig. S1). Dose response studies (using MTT cell viability assays) were performed to determine the physiologically relevant, non-cytotoxic dose range of KTZ at which it inhibited exosome biogenesis and secretion. Figure S1 shows that 1uM KTZ was observed to be non-cytotoxic in the control HEK 293-T as well as three RCC cells. Figure S2 shows the workflow of exosome and EV isolation by DU and sequential ultrafiltration. These secreted exosomes were validated by Immunoblot analyses and tested positive for the expression of tetraspanin (CD63) and exosome biogenesis markers Alix (Fig. 1B). Next, the analysis of secreted exosomes and EVs in conditioned media (collected from RCC-24, 786-O, Caki-2 or HEK 293-T cells treated with KTZ or DMSO for 48 h) was analyzed by the TRPS system (using the NP100 (50–250 nm), Izon nanopore). This exosome quantification indicated that KTZ (1 µM) significantly suppressed exosome secretion in RCC-24, 786-O, and Caki-2 cells, as compared treatment with DMSO (Fig. 1C). Also, exosome quantification using MACSQuant analysis indicated that KTZ (1 µM) significantly suppressed exosome secretion in 786-O-CD63 GFP (Fig. 1D). Interestingly, an increase in secreted exosome was observed in the KTZ treated normal HEK 293-T cells (Fig. 1C). Particle size distribution and diameter was also evaluated and Supplementary Fig. S3 elucidates that there was no significant difference in the particle size-distribution, particle diameter or diameter mode (average size) of exosomes harvested from ketoconazole-treated (1 µM) or DMSO-treated 293-T, RCC24, 786-O, 786-O-CD63 GFP, or Caki-2 cells.

### KTZ functionally inhibits exosome biogenesis and trafficking in RCC cells

**Figure 2:**
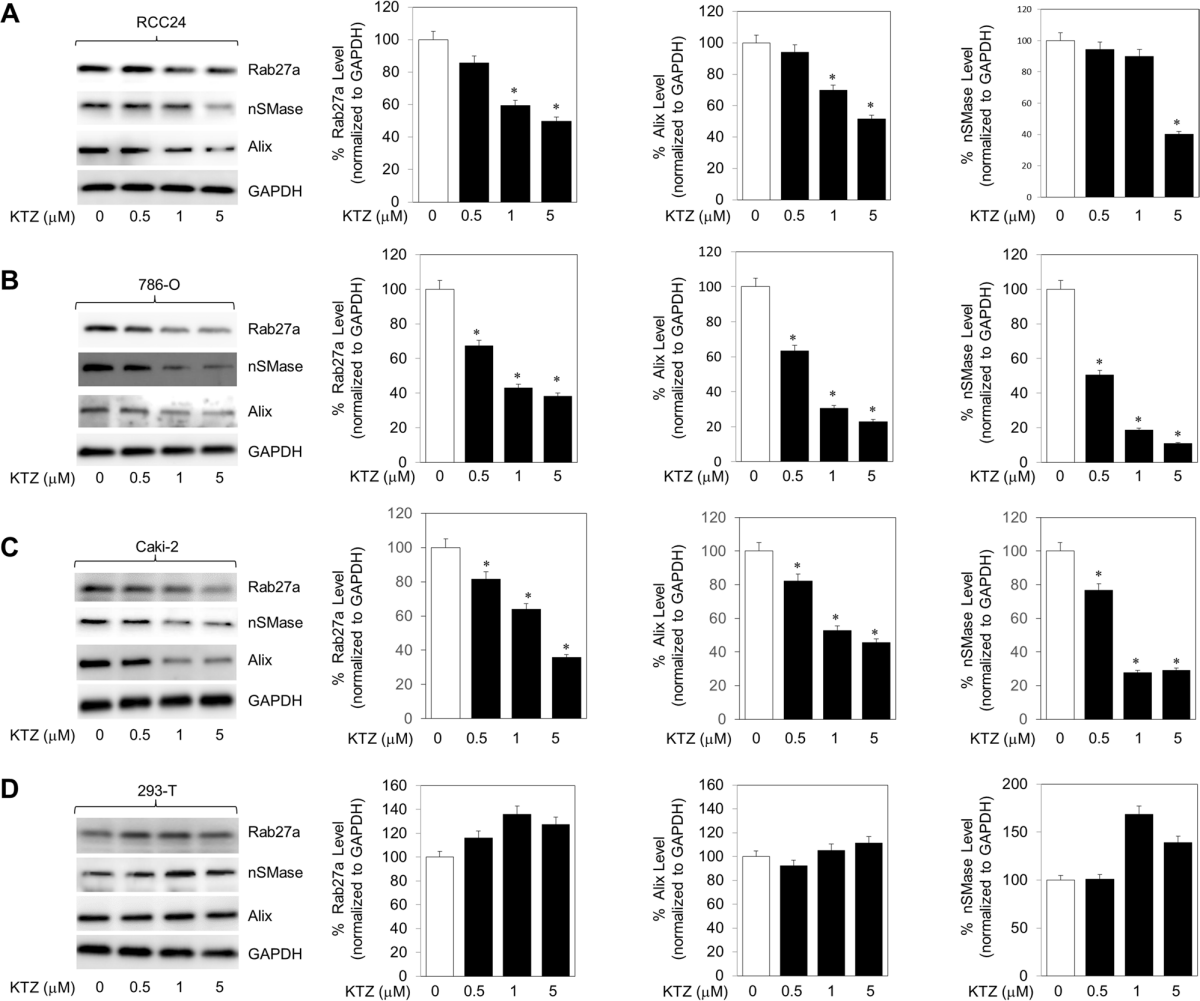
KTZ (prototype imidazole) inhibits exosome biogenesis through ESCRT dependent and independent pathway. KTZ significantly inhibited the protein expression of Alix, nSMase and Rab27a in a dose-dependent manner in RCC-24 (**A**), 786-O (**B**), and Caki-2 (**C**) cells but not in the normal HEK 293-T cells (**D**). Full blots are presented in Supplementary Fig. S6. *Denotes significance at p < 0.05 compared to controls and was calculated using GraphPad Prism.

The endosomal sorting complex required for transport (ESCRT) machinery is pivotal to exosome biogenesis, cargo sorting, and secretion^5,16^. Other ESCRT-independent pathways, i.e., lipid-mediated and tetraspanin-mediated, also play major roles^5,16^. We used 3 separate markers to test KTZ ability to alter pathways associated with exosome transport and biogenesis: Alix, nSMase, and Rab27a. Alix is involved in ESCRT-dependent while nSMase is functional in ESCRT-independent exosomal formation pathway. Rab27a is involved with trafficking of exosomes within the cell. We examined the expression of these markers in cell lines RCC-24, 786-O, Caki-2, and HEK 293-T cells after a 48 h KTZ treatment. A dose-dependent decrease in expression of all markers were observed in RCC-24 (Fig. 2A), 786-O (Fig. 2B), and Caki-2 (Fig. 2C) cells. HEK 293-T cells however did not show a similar dose dependent decrease (Fig. 2D). This suggests KTZ has a cancer-specific mechanism of action.

### KTZ inhibits the ERK signaling pathway in RCC cells

**Figure 3:**
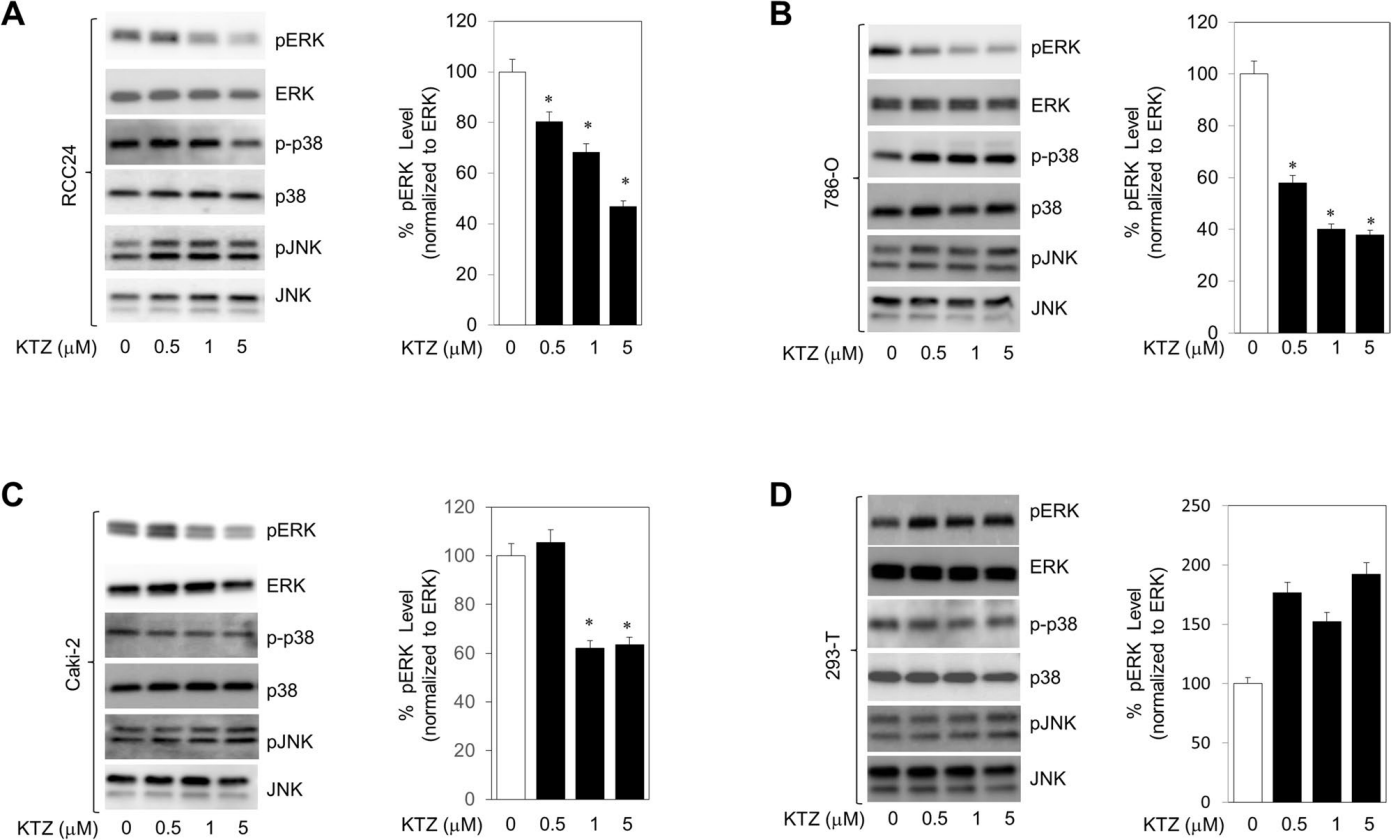
KTZ attenuates exosome biogenesis and secretion through inhibition of the ERK signaling pathway in RCC cells. KTZ significantly inhibited activation p-ERK (downstream effector molecule of the ERK signaling pathway) but not p-p38 or p-JNK in RCC-24 (**A**), 786-O (**B**), and Caki-2 (**C**) cells. Ketoconazole significantly increased activation p-ERK in 293-T (**D**) cells. Full blots are presented in Supplementary Fig. S6. Mean values and standard errors were derived from four independent experiments. *Denotes significance at p < 0.05 compared to controls and was calculated using GraphPad Prism.

In this study, we investigated if KTZ downregulation of MAPKs activation had the potential to inhibit its downstream activators. To do this, we used directed antibodies to the phosphorylated forms of p-ERK, ERK p-p38, p38 p-JNK, and JNK. KTZ treatment showed a significant downregulation of p-ERK expression in RCC-24 (Fig. 3A), 786-O (Fig. 3B), and Caki-2 (Fig. 3C) cells. However, HEK 293-T cells however did not show statistically diminished p-ERK levels (Fig. 3D). We then employed MAPKs pathway selective inhibitors had the ability to alter exosome biogenesis or trafficking. We used Alix, nSMase, and Rab27a expression markers was used to test the function of selective inhibitors U0126, SB203580, SP600125 on exosomes. U0126 inhibits the ERK, SB203580 binds to p38, and SP600125 stops the activation of JNK (Fig. S4). Our ERK inhibitor resulted in a decreased expression of Alix, nSMase, and Rab27a. Both of our p38 and JNK inhibitors were unable to alter the expression of our exosome markers. This is suggestive that KTZ’s ability to diminish exosome formation and trafficking involves the direct attenuation of the ERK signaling pathway (Figs. 3 and S4). In the wider picture, KTZ ability to utilize and inhibit the ERK1/2 pathway in RCC cells increases the anticancer efficacy of sunitinib.

### Effects of KTZ and sunitinib on the proliferation of RCC cells

**Figure 4:**
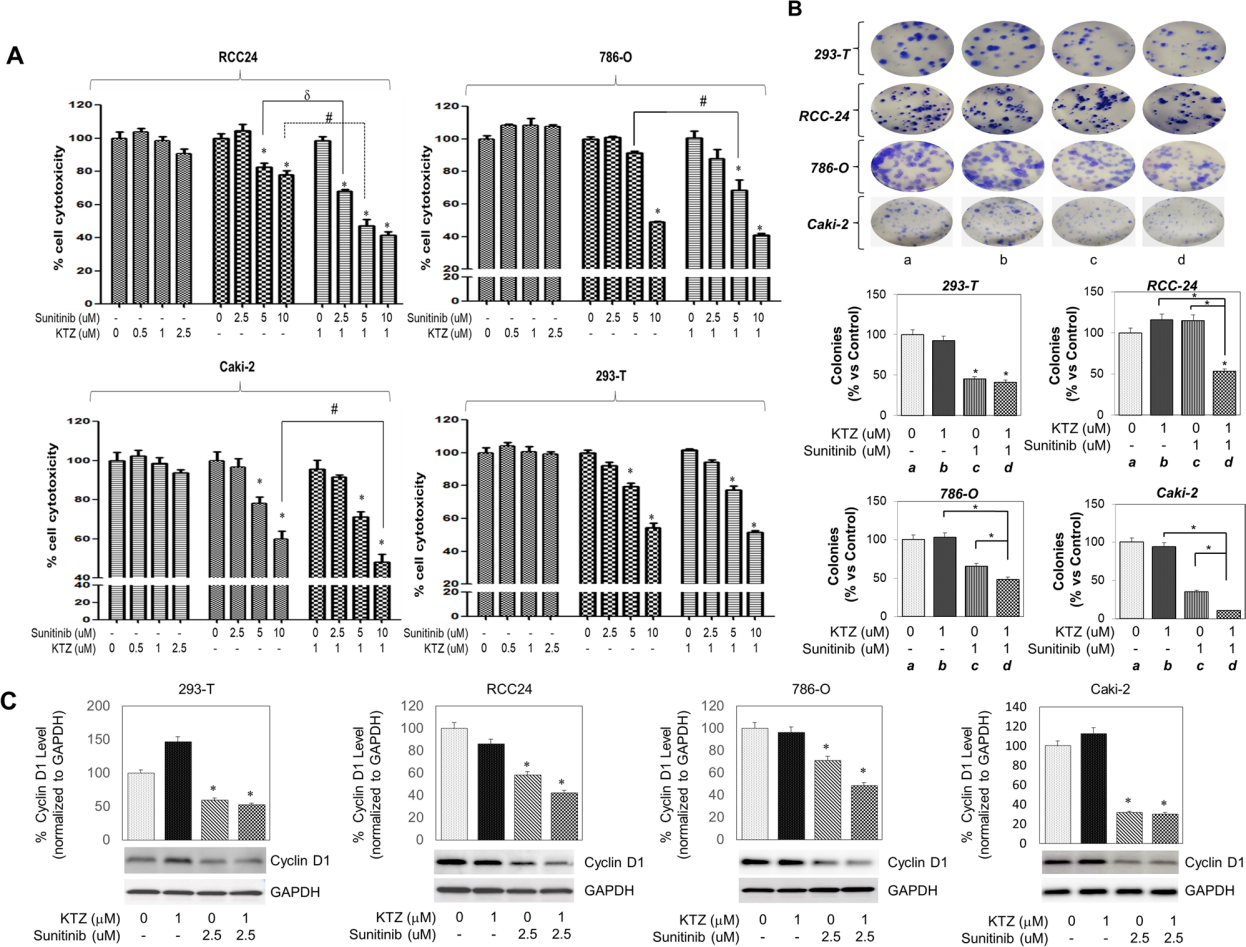
Antiproliferative effects of the two-drug combination are better than those of the single-drug combination in the RCC-24, 786-O and Caki-2 cell lines. (**A**) Combination effect of Ketoconazole and sunitinib on cell proliferation determined by using the MTT assay. Cells were treated with the indicated concentrations of Ketoconazole and sunitinib, and proliferation was determined 48 h later. (**B**) Clonogenic assay was performed in the 293-T, RCC-24, 786-O and Caki-2 cell lines. Colonies were counted 14 days later. Data are presented as the means ± SD from three independent experiments and shown in bar graphs (*p < 0.05 vs. K − K + S or S − K + S). (**C**) Note a significant decrease in cyclin D1 levels after sunitinib treatment and its enhancement in combination treatment with ketoconazole. The densitometric plots shows fold decrease in the expression of signaling molecule. GAPDH immunoblot used as loading control. Full blots are presented in Supplementary Fig. S6.

We then examined the direct effect of the VEGF-targeted tyrosine kinase inhibitor and sunitinib on cell proliferation in both RCC and HEK 293-T cells using MTT assays. As shown in Fig. 4A, MTT assays after sunitinib treatment demonstrated that tumor cell growth was inhibited by sunitinib in all RCC cells (RCC-24, 786-O, and Caki-2) and normal HEK 293-T cells in a dose-dependent manner while KTZ did not show a significant trend. Our investigations clearly showed that combined treatment with sunitinib (5 or 10 µM) resulted in reduction of RCC cells viability. When sunitinib (5 or 10 µM) was used in combination with non-cytotoxic dose of KTZ (1 µM), a significant (P < 0.05) increase in cytotoxicity was observed within 48 h. Combination index (CI) calculations demonstrated that this drug combination functions in a synergistic manner at 48 h post-exposure (CI = 0.34)**.**

### Effects of KTZ and sunitinib on the colony formation of RCC cells

Our investigations also showed that long-term exposure to sunitinib, KTZ, or combination of drugs can suppress the clonogenic ability of RCC cells and further increase the efficacy of ENZ by suppressing CFUs. Although KTZ alone did not significantly decrease CFUs, cells exposed to sunitinib (1 µM) and KTZ (1 µM) combination showed suppression of CFUs (P < 0.05) (Fig. 4B). Thus, KTZ potentiates the efficacy of sunitinib in suppressing the proliferation and clonogenic ability of RCC cells. For cell proliferation, western blot analyses were also performed to determine the downstream genes mediating the proliferation effects of sunitinib, KTZ, or a combination on RCC and HEK 293-T cells. Sunitinib treatment (48 h) of 786-O, RCC24, and Caki-2 tumor cells reduced expression of pro-proliferation genes including cyclin D1, but KTZ treatment did not show a significant decrease in expression of cyclin D1. When sunitinib (2.5 µM) was used in combination with non-cytotoxic dose of KTZ (1 µM) a significant (P < 0.05) decrease in cyclin D1 expression was observed within 48 h, but HEK 293-T cells did not show a significant decrease in between sunitinib or both groups (Fig. 4C). Thus, our results suggested KTZ was synergy effect of decreasing cyclin D1 expression with sunitinib in RCC cells.

### Development of the sunitinib-resistant RCC cell line (786-O-SR)

**Figure 5:**
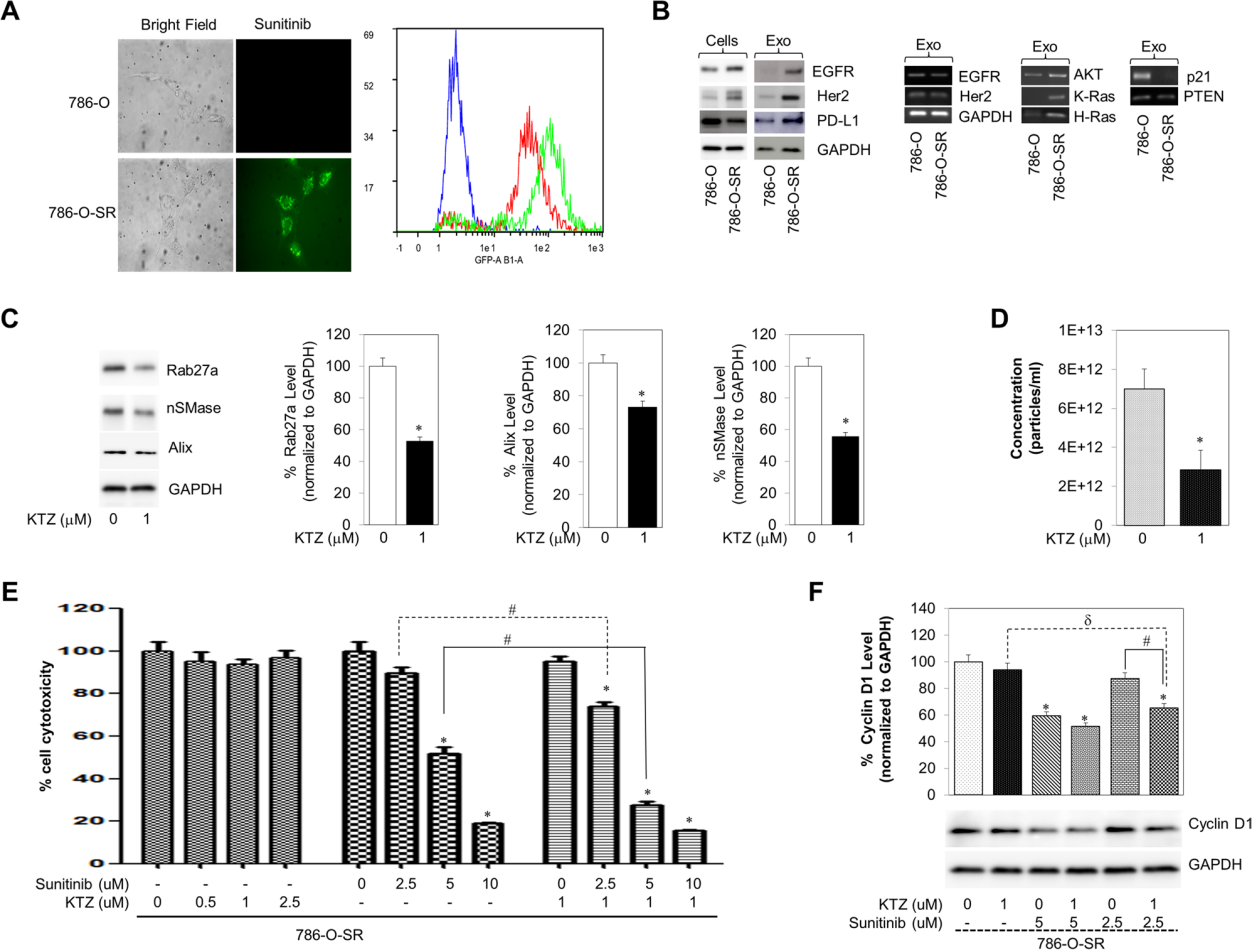
Effect of KTZ co-exposure on 786-O or 786-O-SR cells. (A; left panel) Typical fluorescence microscope image of 786-O-SR cells following treatment with 5 µM sunitinib, exhibiting fluorescent granules (magnification, × 40). (**A**; right panel) Sunitinib autofluorescence in control (Blue) or sunitinib-treated 2.5 μM (Red), and 5 μM (Green) 786-O cells after incubation for 24 h by MACSQuant analysis. This figure was generated using MACSQuantify Software version 2.1.3. This software can be found at https://www.miltenyibiotec.com/US-en/products/macsquantify-software.html#130-094-556. (**B**-Left panel) Total cells and exosome protein lysates were prepared from 786-O and 786-O-SR cells. Proteins were subjected to immunoblot analysis with antibodies against EGFR, Her2, PD-L1, and GAPDH. (**B**-right panel) Conventional PCR analysis of EGFR, Her2, H-Ras, K-Ras and GAPDHs in the exosome derived from 786-O and 786-O-SR cells. (**C**) KTZ significantly inhibited the protein expression of Alix, nSMase and Rab27a in a dose-dependent manner in 786-O-SR. (**D**) qNano-IZON particle quantitative analysis (NP-100 nanopore) depicting a significant decrease in exosome concentrations (50–200 nm size) in the CM of 786-O-SR cells treated with KTZ compared to vehicle treated control. *Denotes significance at p < 0.05 compared to controls and was calculated using GraphPad Prism. **(E)** MTT cell proliferation assays showed that KTZ potentiated sunitinib inhibition of 786-O—Sunitinib Resistance (786-O-SR) cells growth. KTZ synergistically enhanced Sunitinib's cytotoxicity to 786-O-SR cells when the two drugs were combined at the IC50 concentration equivalent for each drug. **(F)** The 786-O-SR cells were treated with the indicated concentrations of sunitinib, ketoconazole or combination drugs for 48 h. Lysates were analyzed by western blot analysis using the indicated antibodies. Relative expression levels of cyclin D1 protein were compared between control cells and cells treated with the indicated drugs by western blot. GAPDH immunoblot used as loading control. Error bars represent SD. Three independent experiments were performed in triplicate. *Denotes significance at p < 0.05 compared to controls. ^d^Denotes significance at p < 0.05 compared between KTZ-treated group and combination-treated group. Full blots are presented in Supplementary Fig. S6. ^#^Denotes significance at p < 0.05 compared between sunitinib-treated group and combination-treated group and was calculated using GraphPad Prism. Data for EVs and exosomes was captured using IZON’s Control Suite software version 3.4.2.48, https://support.izon.com/how-can-i-get-the-latest-software-release.

Sunitinib is a mainstay for treatment of advanced RCC patients^17^. However, up to 20% of advanced RCC patients are inherently refractory to sunitinib therapy, and almost all remaining patients develop drug resistance and tumor progression within 15 months of therapy^18^. Thus, it is imperative to elucidate the underlying mechanisms of sunitinib resistance in RCC patients and those of other advanced therapeutic drugs as well. Sunitinib has the ability to become autofluorescent and absorbs light at a wavelength from 340–480 nm with 429 nm exhibiting peak absorption. Sunitinib reflects light with wavelength, at maximum, of 540 nm. Under fluorescence microscopy, green fluorescent sunitinib was enclosed within intracellular granules (Fig. 5A; left panel). Subsequently, MACSQuant analysis was conducted to follow the dynamic accumulation of 2.5, or 5 µM sunitinib in living cells. The results revealed that 24 h after adding sunitinib, the cells displayed clear green fluorescence (Fig. 5A; Right panel). To evaluate the median toxic dose (TD50) of KTZ and sunitinib, we employed cell viability studies individually on 786-O and 768-O-SR cell lines (Fig. S5A,B).

### Effects of KTZ inhibit sunitinib resistance cancer cell proliferation and modulate onco-protein and mRNA delivery of 786-O-SR exosome

Exosome released by cancer cells can contain elevated levels of oncogenic factors that can recruit surrounding cells^6–10^. Recently, Qu and colleagues^2^ suggested that IncARSR embedded in exosomes derived from sunitinib-resistant cells could confer the resistant phenotype to recipient RCC cells. To determine the oncogenic potential of 786-O-SR exosome, we measured the transcript levels of several oncogenes, and immune resistance gene in exosome isolated from 786-O and 786-O-SR cells. 786-O-SR exosome contained more mRNA, or protein for the oncogenes AKT, KRAS, HRAS, EGFR, and Her2, the tumor suppressor genes p21, and PTEN, and the immune resistance gene PD-L1 compared with exosome isolated from 786-O cells (Fig. 5B). Next, we examined key regulatory cellular targets involved in biogenesis and secretion in KTZ treated 786-O-SR cells. KTZ treatment (48 h) in 786-O-SR cells reduced expression of Alix, nSMase and Rab27a, as evidenced by immunoblot (bottom panels) analyses (Fig. 5C). The pathways involved in the biogenesis and secretion of exosome (50–250 nm), particle measurements were carried out to determine if their size-distribution and concentrations are modulated by the KTZ treatment in 786-O-SR cells. Supplementary Figure S2 shows the workflow of exosome isolation by DU and sequential ultrafiltration. Analysis of secreted exosome in the CM of 786-O-SR cells treated with KTZ or DMSO for 48 h was performed by the TRPS system using NP100 (50–200) nanopores. Exosome quantification using NP100 nanopore (Izon) indicated that KTZ (1 µM) significantly suppressed exosome secretion in 786-O-SR by ~ 60%, as compared to the controls (Fig. 5D). The two drugs exhibited a dose‐dependent growth inhibition of 786-O-SR cells, as shown in Fig. 5E. It has been well demonstrated that the combination therapy of KTZ and sunitinib also exhibited a dose‐dependent growth inhibition of 786-O-SR cells and Cyclin D1 (Fig. 5F). These results suggested KTZ was synergy effect of decreasing cyclin D1 expression with sunitinib in RCC cells. Finally, our results demonstrated that KTZ in renal malignancies play a role in sunitinib resistance or sensitive and that inhibition of these exosome may delay or prevent the development of resistant tumors.

## Discussion

Current estimates place the cost of bringing new medications through federal drug administration (FDA) approval at more than 1 billion dollars with an optimistic timeline of 10 years. Thus, drug repurposing provides an ideal mechanism for novel therapeutics or adjunct therapies. By identifying medications which have been proven safe in humans and then utilizing their unanticipated or previously unrecognized side effects, bypassing the drug discovery could create new therapeutics without the hassle of drug creation and safety evaluation. Utilization of ketoconazole in its role of exosome inhibition produces unique opportunities for improved cancer therapy without expense of drug development.

Ketoconazole, a broad-spectrum antifungal agent derived from imidazole, has been used previously for off label purposing including its CYP17 inhibition and causation of near castrate level testosterone in prostate cancer patients^19^. Growing evidence suggests that ketoconazole alone or in combination with other agents may possess considerable antitumor properties including prostate^20^, breast^21^, melanoma^22^, and hepatocellular carcinoma^23^. It is important to highlight ketoconazole’s ability to inhibit cytochrome-P450 enzymes types. When ketoconazole is used in combination of other drugs, it has the potential to increase the Cmax of the drug concurrently being administered. Moreover, the oral use of ketoconazole is well tolerated in cancer patients with limited toxicity in several clinical studies^24,25^. No studies have evaluated KTZ in the treatment of RCC. In prostate cancer studies, we have documented that KTZ effectively and reproducibly inhibits exosome biogenesis in a dose dependent fashion lines^13^. Exosome biogenesis is initiated within the endosomal system and the ESCRT machinery is important for the process of endosomal membrane invagination. We identify alterations in Alix, nSMase, and Rab27a inhibition in the RCC cell lines, but not the control HEK293. We showed that a potent inhibition of exosome biogenesis was possible using non-toxic and physiologic dosing of KTZ. Furthermore, we observed that KTZ led to suppression of multiple pathways leading to exosome biogenesis and secretion. Thus, KTZ having lesser side effects but possessing high exosome inhibitory effects may be repositioned as anti-cancer agents.

The incidence of RCC has been rising through the world^26^. Since the 2000s, there have been dramatic advances in the treatment of metastatic renal cell carcinoma (mRCC), including drugs targeting, such as the oral, multi-targeted receptor tyrosine kinase inhibitors (TKIs) sunitinib^18,27^ and sorafenib^27,28^, and mammalian target of rapamycin (mTOR) inhibitors temsirolimus^29^ and everolimus^30^ pathways. Recently, the newer VEGR inhibitors, pazopanib^31^ and axitinib^27^ have also demonstrated efficacy in the treatment of patients with mRCC. However, approximately 20% of advanced RCC patients are inherently refractory to sunitinib therapy, and almost all the remaining develop drug resistance and tumor progression within 15 months of therapy^17^. This leads to limited increase in overall life expectancy. It is necessary to investigate the biological basis of sunitinib resistance and identify novel targets for sunitinib-resistance prevention and therapy. Additionally, there are two types of drug resistance: Primary and Secondary. Primary drug resistance is characterized by patients who do not initially respond to pharmacological therapy. Secondary drug resistance is defined as patients who relapse or progress after initially responding to medical treatment. In this study, we seek to answer mechanisms surrounding secondary sunitinib resistance. We believe that our drug combination therapy has the potential to address primary drug resistance, through decreasing exosome biogenesis and secretion, but further investigation is needed to fully elucidate this concept.

Currently, exosomes in the cancer cell environment provide extracellular information transmission and drug resistance. Identifying novel mechanism for inhibition of exosomal transfer could be paramount to the success of cancer therapies^6,8,18,32,33^. As exosomes are known to transmit cargos including miRNA, lncRNA, along with other mechanism for chemoresistance, the inhibition of their secretion or biogenesis may provide novel ancillary targets for adjunctive therapy^2,3,8,34,35^. It has been established that in RCC, exosomes play a role in sunitinib resistance. Qu et al.^2^ also found that lncARSR could be secreted from resistant cells via exosome, transforming sunitinib-sensitive cells into resistant cells, thereby disseminating drug resistance. Other research has demonstrated the role of the exosomes in tumoral cross talk with immune cells^36^. Yang et al*.* showed that exosomes from kidney adenocarcinoma cells contain Fas ligand and trigger Jurkat T cell apoptosis, contributing to the immune evasion of tumors^37^. In our data, KTZ inhibited exosome biogenesis and secretion through inhibition of protein expression of Alix, nSMase, and Rab27a related to exosome biogenesis and secretion in RCC cells. In 786-O-SR cells, treatment with KTZ alone inhibited the growth of cancer cells through inhibition of cyclin D1 protein expression, and the combination of sunitinib and KTZ increased the anticancer effect. Novel antitumor therapy strategies based on exosomal inhibition in have significant implications in the tumor microenvironment and in cancer therapy. If safe and efficacious, in the future for the treatment of RCC we might consider a lower dose sunitinib in combination with ketoconazole.

It must be noted that the metabolism of Sunitinib is mediated by Cytochrome P450 3A4 (3A4). Detoxification by 3A4 is predominantly done within the liver and, to a lesser extent, kidneys. Ketoconazole is a well-established inhibitor of 3A4. The in-vivo bioavailability of Sunitinib when co-administrated with Ketoconazole was described by Chee et al.^38^ After co-administration, an increase of sunitinib’s AUC_plasma_ was seen. However, the dule drug’s AUC_tissue_ to AUC_plasma_ ratio within the kidney was found to be comparable to Sunitinib treatment alone. Chee et al. hypothesized that this increased AUC_plasma_ could be attributed to an altered liver first-pass metabolism by 3A4. The role of 3A4 inhibition within in-vitro kidney cancer models is still poorly understood. By inhibiting renal 3A4 through Ketoconazole, it is possible sunitinib would have displayed higher bioavailability and potentially confounded the synergic findings of the two medications in this study. According to our data, ketoconazole functions to alter exosome concentrations, exosome biogenesis/trafficking pathways, pERK expression, and cyclin D1 levels. The alteration of exosomes enables sunitinib to decrease the viability in both suni-sensitive and -resistant cell lines. Exosomes were of particular focus because functional resistance to sunitinib has been shown to be carried by these small lipid vesicles. However, the inhibition of 3A4 by ketoconazole has the potential to be an alternative route to affect sunitinib. 3A4 was not the focus of this present study; further research into this additional pathway and its relation to exosomes is currently underway.

**Figure 6:**
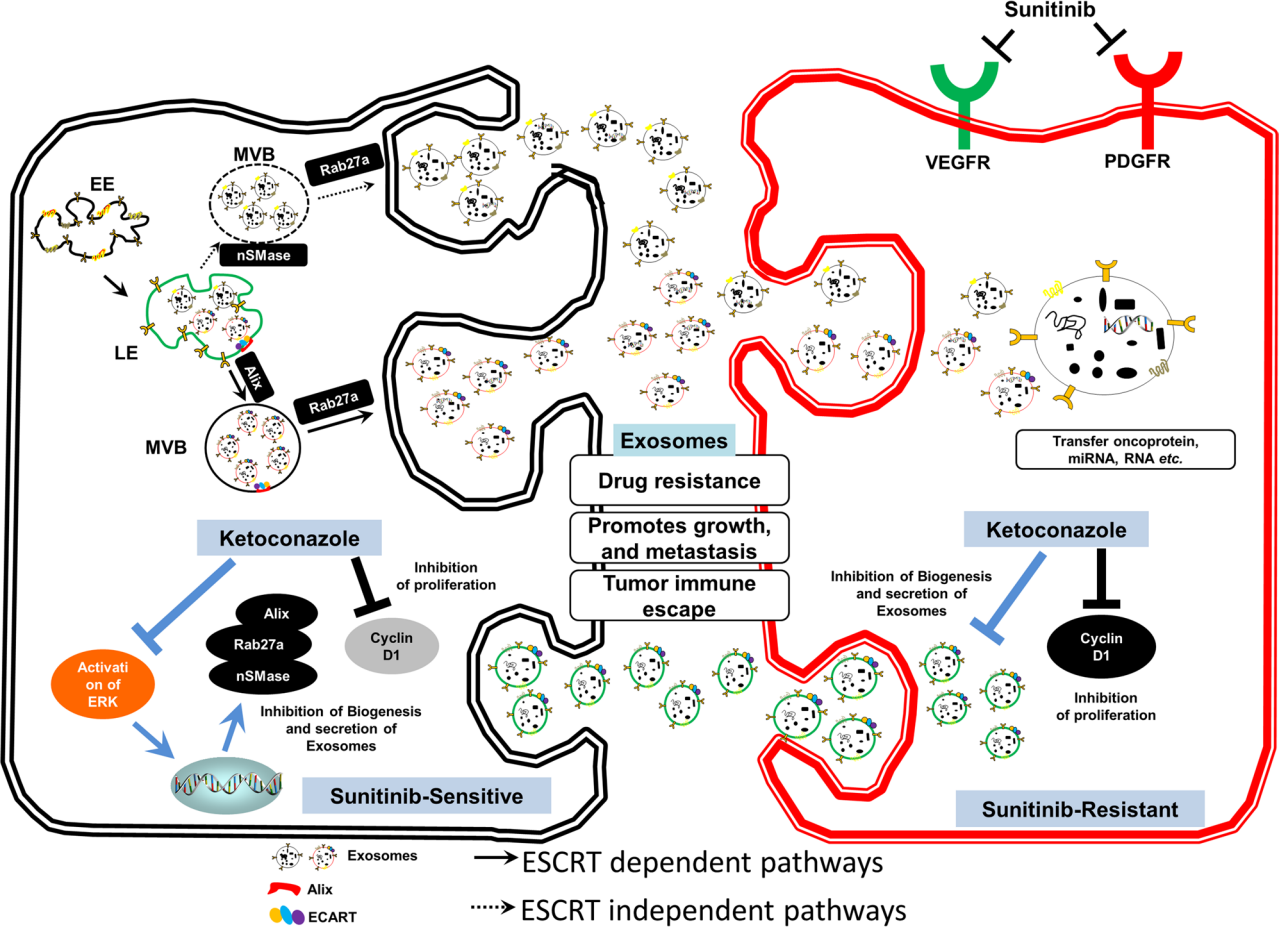
Illustrative diagram for exosome-mediated transfer of therapeutic resistance in the RCC microenvironment by the clinically approved KTZ. Exosome act as intercellular messengers that give the ability to communicate between both cells of the same type and other cell types. The exosome cargoes contain nucleic acids (miRNAs, DNAs, and RNAs), proteins (cytoplasmic proteins, tetraspanins, and membrane receptors), and lipids (ceramides, and cholesterol). The exosomes secreted from cancer cells can affect the local tumor microenvironment, alter the extracellular matrix, and enhance the drug resistance, cancer cell growth, metastasis, and immune escape. The initial steps of this process are usually modulated by the endosomal sorting complex required for transport (ESCRT) dependent or ESCRT independent pathway, and then the mechanisms involved in the release of exosomes are also regulated by other protein families, such as Rab GTPases such as Rab27a and Rab27b. The inhibition of ERK1/2 pathway by KTZ leads to transcriptional downregulation of Alix, nSMase, and Rab27a. A inhibition of exosome biogenesis and secretion relative proteins, and the inhibition of cyclin D1 by KTZ are decreased the tumor growth of RCC as well as sunitinib resistant cancer cells. (EE) early endosome; (LE), late endosome; (MVB), multivesicular bodies; (ESCRT), endosomal sorting complex required for transport.

We believe the dual therapy of Ketoconazole and Sunitinib is effective in the pre-clinical setting to decrease tumor specific exosome (Fig. 6). However, a randomized controlled clinical trial is needed to validate if the ketoconazole + sunitinib treatment is safe and effaceable in the clinical setting. Furthermore, additional analysis of this data is required to validate the therapeutic benefit. Future studies will focus on the efficacy in small animal models for renal malignancy prior to translation of this work into humans.

## Material and methods

All experimental protocols were approved by an Intuitional Review Board (IRB) and patient consent was obtained for the acquisition of all human samples. Approval number IRB #2017-483-TUHSC was obtained from the Tulane University Health Sciences Center (TUHSC), Human Research Protection Office & Intuitional Review Board. All methods were carried out in accordance with relevant guidelines and regulations.

### Materials

RPMI 1640, penicillin/streptomycin solution, fetal bovine serum (FBS) were obtained from Invitrogen (Camarillo, CA, USA). Ketoconazole (KTZ) and sunitinib were purchased from Selleckchem (Houston, TX). U0126 and SB203580 were procured from Promega (Madison, WI, USA) and SP600125 from Sigma (Sigma-Aldrich, St. Louis, MO, USA). Unless otherwise indicated, all other drugs were purchased from Sigma (St. Louis, MO, USA)^11,12^.

### Patients and plasma sample preparation

The 59 plasma samples of kidney cancer patients were procured from the Biospecimen Core Laboratory (BCL) of the Louisiana Cancer Research Center (LCRC) (New Orleans, LA, USA). These samples were collected from subjects with tumors of various pathological stages including pT1 and pT3. Venipuncture blood was collected in EDTA tubes and then centrifuged for 15 min at 2000×*g* at 4 °C. Plasma was aliquoted and stored at − 80 °C.

### Cell culture and plasmids

Renal cancer cell lines (786-O, Caki-2) and the human embryonic kidney cell line, 293-T, were purchased from ATCC (Manassas, VA, USA). The RCC24 cells were provided by Dr. Arnold Zea, LSUHSC, New Orleans. The RCC cells were cultured in RPMI 1640 medium supplemented with 10% fetal bovine serum, 2 mM L-glutamine, and 1% penicillin/streptomycin (P/S). Moreover, for routine maintenance, each cell line was cultured as a monolayer at 37 °C in a 5% CO2, 95% air incubator^11^**.** To induce resistance, the sunitinib-resistant sub-lines 786-O-SR (Sunitinib Resistance), continuously cultured in the presence of 5 μM sunitinib has been described previously^39^. Following continuous culture in the complete medium supplemented with 5 µM sunitinib for more than 10 passages. The plasmid (CD63-GFP) (Origene cat#RG201733) was used to generate the stable 786-O-CD63-GFP cells^13^. For collection of conditioned media (CM), the cells were trypsinized, plated, and allowed to attach overnight in complete media. Following overnight growth, the media was changed to FBS-free media for 48 h and the supernatant was collected for exosome isolation^12^.

### Exosome isolation

Exosome from conditioned media (CM) of RCC cells cultured in exosome-free media containing Ketoconazole or control vehicle were purified by differential ultracentrifugation (DU) as we described^11–13^ with minor modifications.

### Analysis of exosome particles by the qNano IZON system

As we described previously^11–13^, we employed the TRPS technology (qNano IZON system; Izon, Cambridge, MA, USA) to measure the concentrations, size-distribution, and diameters of the exosome in the CM of RCC and 786-O-CD63 GFP cells treated with the Ketoconazole (KTZ) or control vehicle (DMSO). The system was calibrated for voltage, stretch, pressure, and baseline current using two standard beads: CPC100B (mode diameter: 114 nm, concentration: 1.0E13/ml) and CPC70D (mode diameter: 70 nm, concentration: 3.0E13/ml). A diluted sample size of 40 μL and NP100 nanopore (for 50–200 nm size range) were used and data analysis was performed by a qNano IZON Control Suite software^40^.

### Analysis of exosome by flow cytometry

Exosome derived from CM of 786-O-CD63-GFP cells were cultured in serum-free media and treated with the drug candidates or DMSO for flow cytometry analysis. The MACSQuant Analyzer 10 system (Miltenyi Biotec Inc., San Diego, CA, USA) configured with Forward (FSC) and Side Scatter (SSC) channels (488 nm laser) that include fluorescent channels with detection excitations from 405 nm, 488 nm, and 638 nm lasers. Optimization was performed to yield the best experimental conditions with FSC and SSC, triggers set to detect only EVs with maximally reduced background noise. In short, for the optimized detection of EV’s, an SSC trigger was set to reduce electronic noise using non EV containing PBS filtered through a 0.22 μm Millex-HV Syringe Filter (EMD Millipore, Billerica, MA, USA) and set as background noise for the patient sample EVs. A fluorescent trigger was also used on the B1 channel which has a 488 nm excitation and a bandpass filter allowing 525/50 nm wavelengths of emitted light. This allowed further reduction of electronic noise. Nano Fluorescent standard particles (Cat. No. NFPPS-52-4 K, Spherotech Inc. Lake Forest, IL, USA) were used as a reference standard to calibrate the experiment based on particle size and fluorescence. The MACSQuant Analyzer 10 syringe driven fluidics system allows for the volumetric measurement of samples that can be quantified on an events/µL or events/mL basis. Methods for exosome by flow cytometry analysis were previously described^11,12^ with minor modifications.

### MTT assay

Cell viability was measured by the MTT (methylthiazolyldiphenyl-tetrazolium bromide) (Sigma-Aldrich) cell cytotoxicity assay according to the manufacturer’s protocol as we described^12,41–43^. Briefly, approximately 5,000 cells were seeded in 96-well cell culture plates and allowed to adhere overnight. Cells were then synchronized by overnight incubation in serum-free medium and treated with desired concentrations of drug(s) and controls for 48 h. Cell viability was determined by incubating the treated cells with MTT solution (5 mg/ml) for 1 h at 37 °C. DMSO was then added to the wells, and the optical density (O.D.) of formazan crystals solubilized in DMSO was measured at 570 nm by using a µQuant spectrophotometric plate reader from Bio-Tek (Seattle, WA, USA). In each individual experiment, changes in cell survival following drug treatments are expressed as percent of untreated control.

### PCR and immunoblot analyses

Oligonucleotide primers for convention PCR was synthesized by Integrated DNA technologies (Coralville, Iowa) and are listed in Table S1. Total RNA and proteins were isolated from various using standard protocols as we described^11^. Total RNA was subjected to conventional PCR analyses using a master mix from Bio-Rad Laboratories (Hercules, CA). Protein extracts were subjected to immunoblot analysis using antibodies against PD-L1, EGFR, Alix, p-ERK, ERK, p-JNK, JNK, p-p38, p38, cyclin D1 (Cell Signaling Technology, Danvers, MA, USA), CD63, Her2 and GAPDH (Santa Cruz Biotechnology, Dallas, TX), Rab27a (Proteintech, Chicago, IL, USA) and nSMase (Abcam, San Francisco, CA, USA). Immune complexes were detected with appropriate secondary antibodies from Jackson ImminoResearch Inc. (West Grove, PA, USA) and chemiluminescence reagents (Bio-Rad, Hercules, CA, USA) as described^13,44^. Immunoblot signals were captured using the Image Quant Las 300 (GE Healthcare, Piscataway, NJ, USA). Densitometric analysis was performed using ImageJ (NIH, Bethesda, MD, USA, http://imagej.nih.gov/ij/).

### Colony forming units assay

Colony forming units assay was performed using protocols previously described^11,41^. RCC cells (500 cells/dish) were seeded in 60-mm petri dishes in 3 replicates and grown in RPMI 1640 medium supplemented with 2% FBS. The drugs were added after 48 h and replenished in the second week. After two weeks in culture, colonies were fixed with 100% ethanol and stained with 0.2% crystal violet in 20% methanol. The colony forming units (CFU) were enumerated using ImageJ software (NIH). Change in total CFUs drug-treated cultures were compared to the control (untreated) cultures.

### Statistical analysis

Data are presented as Means ± S.E.M. of more than three independent experiments performed in triplicate. For Western blots, a case representative experiment is depicted in the figures section. Comparisons between multiple groups were performed with ANOVA with Bonferroni’s test using GraphPad Prism. Data were considered statistically significant at *P* < 0.05 as previously described^11,12^.

## Supplementary Information

Supplementary Information.
