# Supplementary material for: Repurposing ketoconazole as an exosome directed adjunct to sunitinib in treating renal cell carcinoma
Source: Sci Rep. 2021 May 13;11:10200. doi: 10.1038/s41598-021-89655-w (PMC8119955; doi:10.1038/s41598-021-89655-w)
Supplement: Supplementary file 1 — Supplementary Information. [file 41598_2021_89655_MOESM1_ESM.pdf]

Supplementary Data

Repurposing Ketoconazole as an Exosome Directed Adjunct to Sunitinib in treating Renal Cell Carcinoma

Jacob W. Greenberg[1,#], Hogyoung Kim[1,#,*], Ahmed A. Moustafa[1,6], Amrita Datta[1,4], Pedro Barata[3], A. Hamid Boulares[5], Asim B. Abdel-Mageed[1, 2, 4], and Louis S. Krane[1,4,δ]

[1]Departments of Urology and [2]Pharmacology, and [3]Department of Internal Medicine, Section of Hematology/Oncology, Tulane University School of Medicine, New Orleans, LA 70012.[4]College of Nursing and Health, Loyola University New Orleans, LA 70118. [5]The Stanley Scott Cancer Center/Louisiana Cancer Research Center, School of Medicine, Louisiana State University Health Sciences Center, New Orleans, LA, USA. [6]Zoology and Entomology Department, Faculty of Science, Helwan University, Cairo 11790, Egypt.

Figure S1

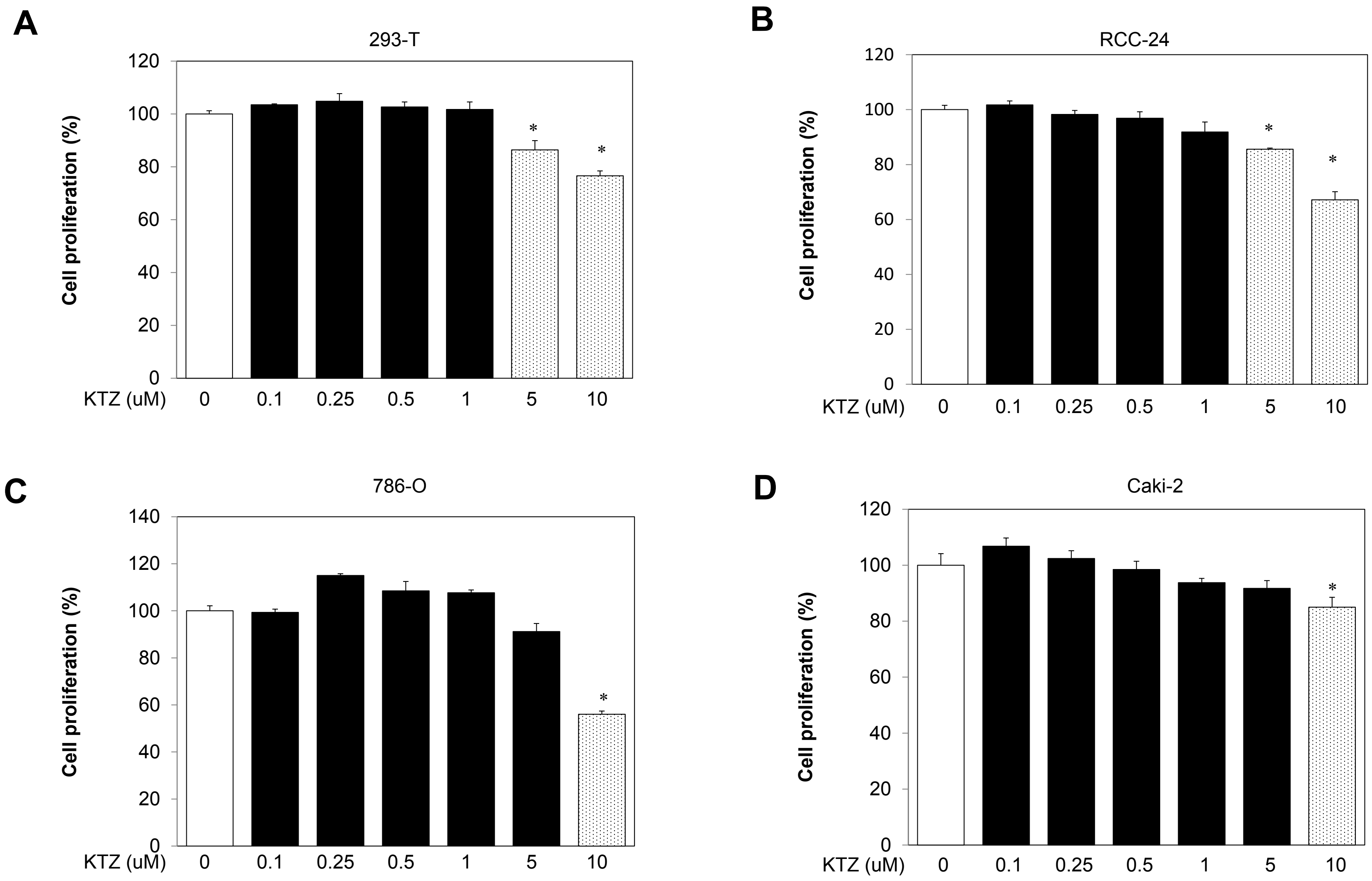

Fig S1: Effect of KTZ on cell viability. HEK 293-T (A), RCC24 (B), 786-O (C) and Caki-2 (D) cells were treated with vehicle (0;DMSO) or a dose dependent manner of KTZ for 48 h. Viable cells were measured by MTT assay (*n*=3). Data were analyzed by one-way ANOVA followed by Bonferroni post-tests. *$p<0.05$, vs. control.

Figure S2

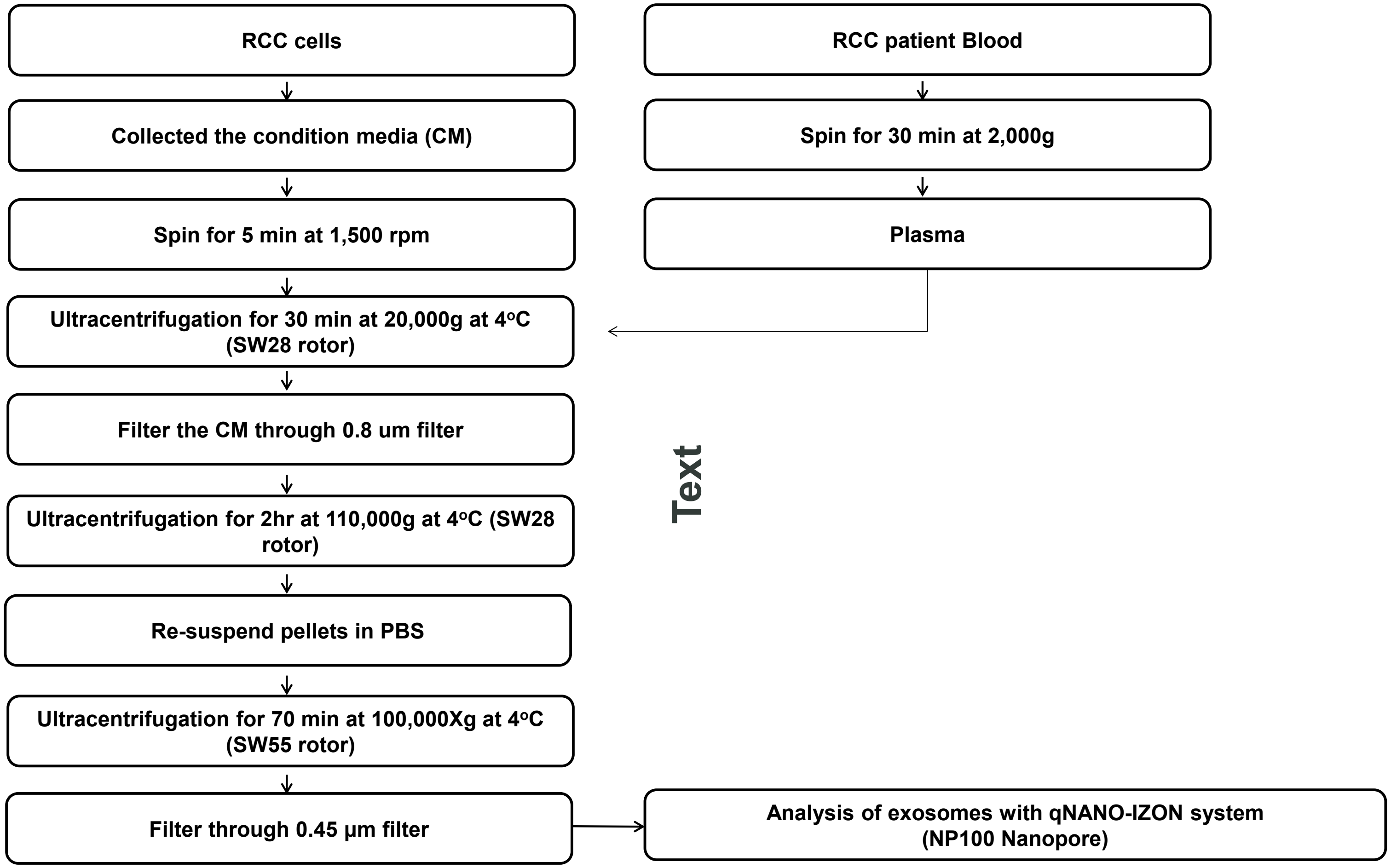

Fig S2: Flowchart of isolation and analysis of the extracellular vesicles (EVs). Purification and analysis of EVs including exosomes in the conditioned media (CM) of RCC cells treated with DMSO (vehicle) or KTZ (1 µM) were performed according to the depicted flow chart. Following differential ultracentrifugation, MVs were prepared by filtration through 0.8 µm and 0.45 µm filters, respectively, and analyzed by qNano-IZON system using NP100 (size range: 50-200 nm) nanopore, respectively.

Figure S3

			Mode	St Dev			Mean
**A**	293-T	DMSO	111	40.9	HEK293-T	DMSO	88
		1 μM KTZ	109	37.5		1 μM KTZ	84
			Mean	St Dev			Mean
**B**	RCC24	DMSO	129	47.1	RCC24	DMSO	98
		1 μM KTZ	124	43.7		1 μM KTZ	96
			Mean	St Dev			Mean
**C**	786-O	DMSO	108	34.7	786-O	DMSO	91
		1 μM KTZ	103	35.1		1 μM KTZ	79
			Mean	St Dev			Mean
**D**	Caki-2	DMSO	107	35.8	Caki-2	DMSO	86
		1 μM KTZ	114	44.4		1 μM KTZ	93

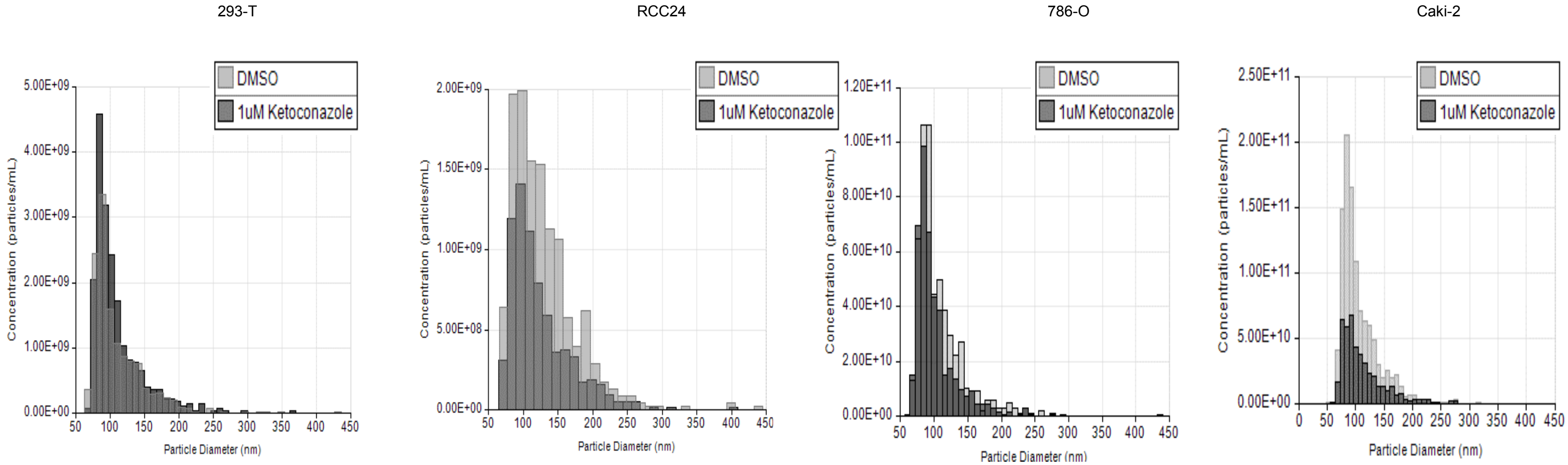

Fig. S3 Effect of ketoconazole on the diameter of RCC cell-secreted EVs. 293-T **(A)**, RCC24 **(B)**, 786-O **(C)** or Caki-2 **(D)** cells were treated with ketoconazole (1 uM) or DMSO at different time intervals and particle diameter and diameter mode of exosomes were measured with qNano-IZON system. There was no significant difference in the exosome diameter or diameter mode of exosomes harvested from ketoconazole-treated or DMSO-treated 293-T, RCC24, 786-O or Caki-2 cells. Particle size distribution figures were made using IZON's Control Suite Software version 3.4.2.48. This software can be found at https://support.izon.com/how-can-i-get-the-latest-software-release.

Figure S4

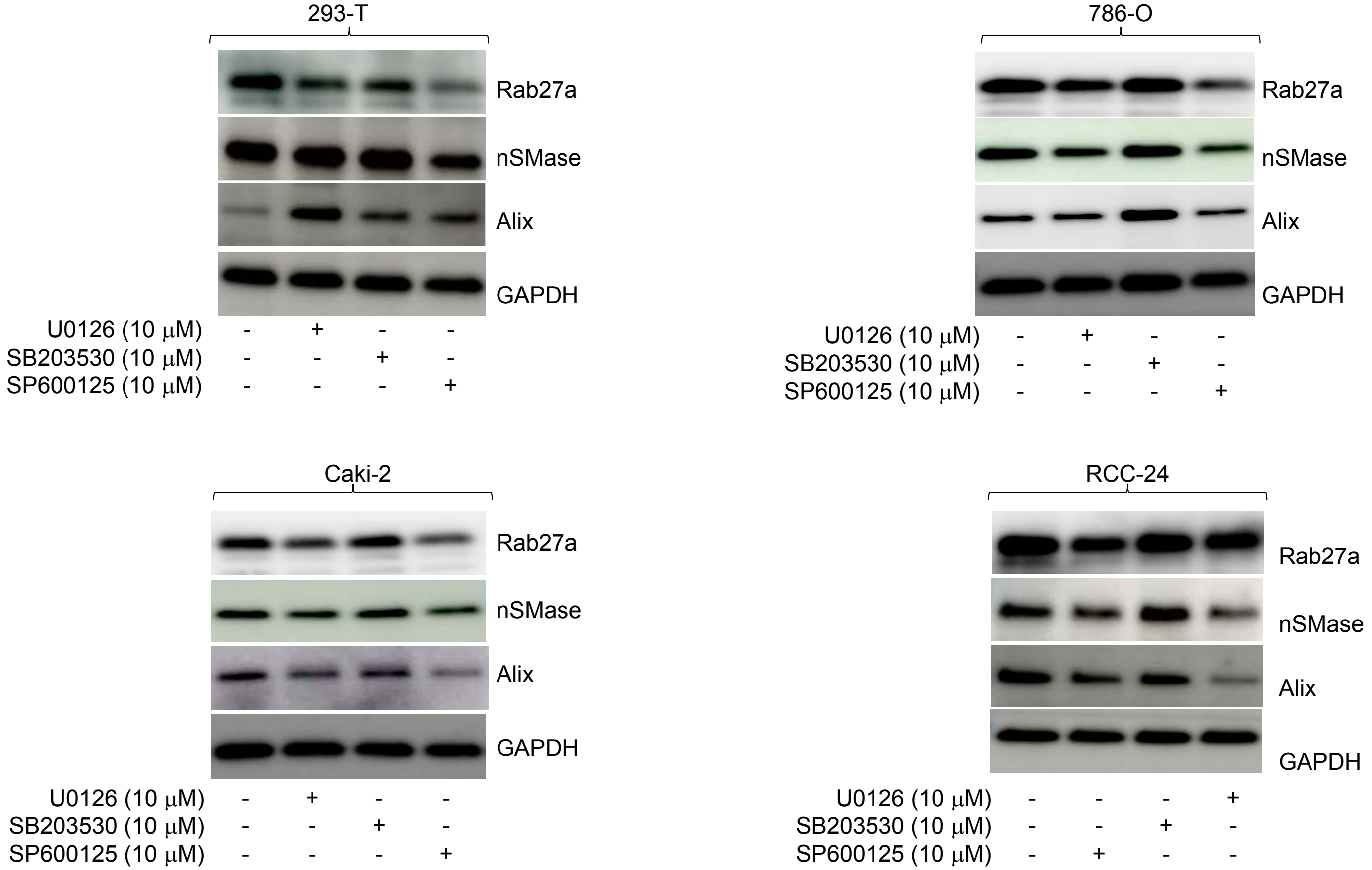

Fig. S4 Effects of MAPK inhibitors on Rab27a, nSMase and Alix protein expression. Cells were treated with inhibitors(10uM); inhibitor for MEK1/2 and ERK1/2, SB202190, inhibitor for p38MAPK, and SP600125, inhibitor for JNK. (means ± SEM of four independent experiments).

Figure S5

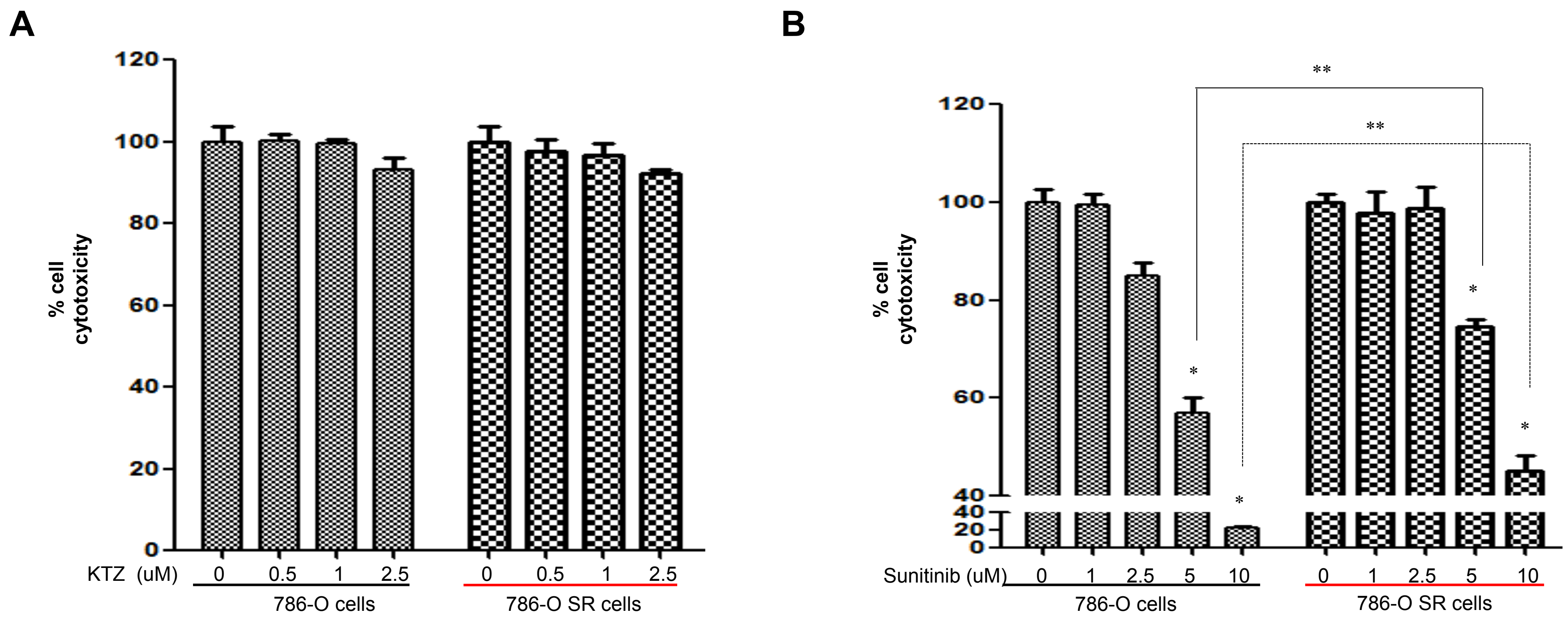

Fig. S5 Cytotoxic effects of a dose dependent concentration of KTZ are shown in 786-O and 786-O-SR cells following 48 h (n=3).

Figure S6-1

Full blot images across all figure

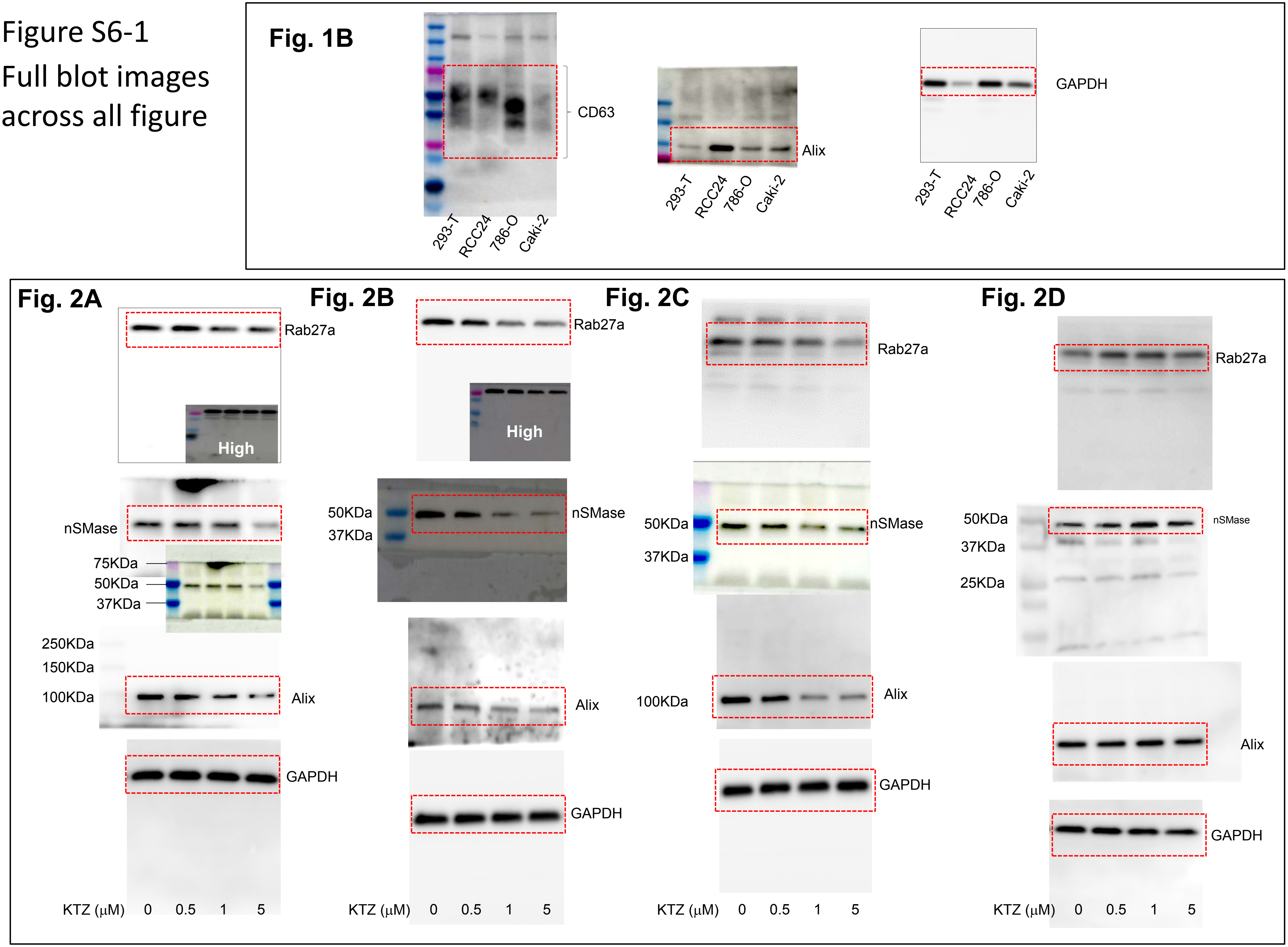

Figure S6-2

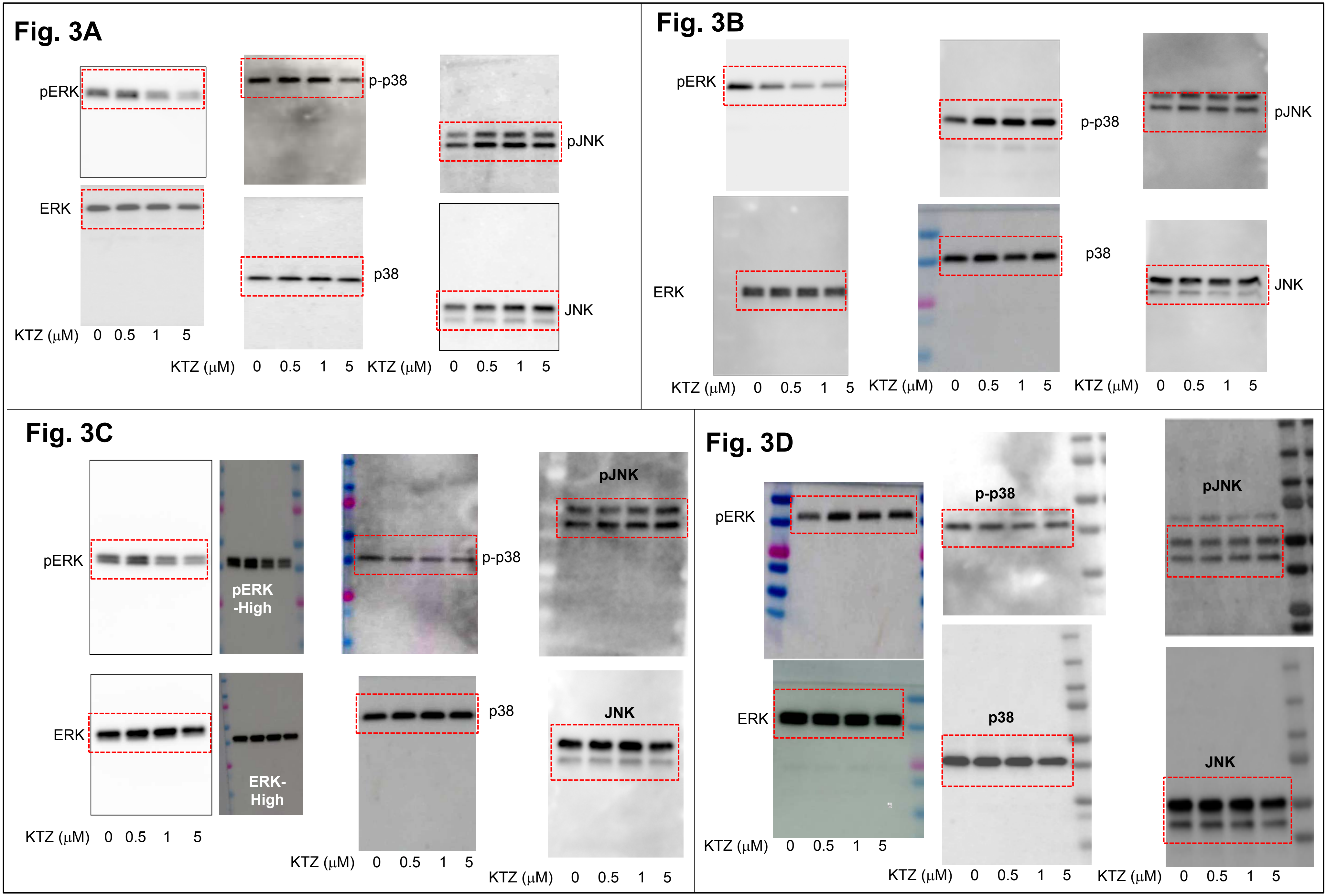

Fig. 3A
pERK
ERK
KTZ (μM) 0 0.5 1 5
p-p38
p38
KTZ (μM) 0 0.5 1 5
pJNK
JNK
KTZ (μM) 0 0.5 1 5
Fig. 3B
pERK
ERK
KTZ (μM) 0 0.5 1 5
p-p38
p38
KTZ (μM) 0 0.5 1 5
pJNK
JNK
KTZ (μM) 0 0.5 1 5
Fig. 3C
pERK
pERK -High
ERK
ERK- High
KTZ (μM) 0 0.5 1 5
p-p38
p38
KTZ (μM) 0 0.5 1 5
pJNK
JNK
KTZ (μM) 0 0.5 1 5
Fig. 3D
pERK
ERK
KTZ (μM) 0 0.5 1 5
p-p38
p38
KTZ (μM) 0 0.5 1 5
pJNK
JNK
KTZ (μM) 0 0.5 1 5

Figure S6-3

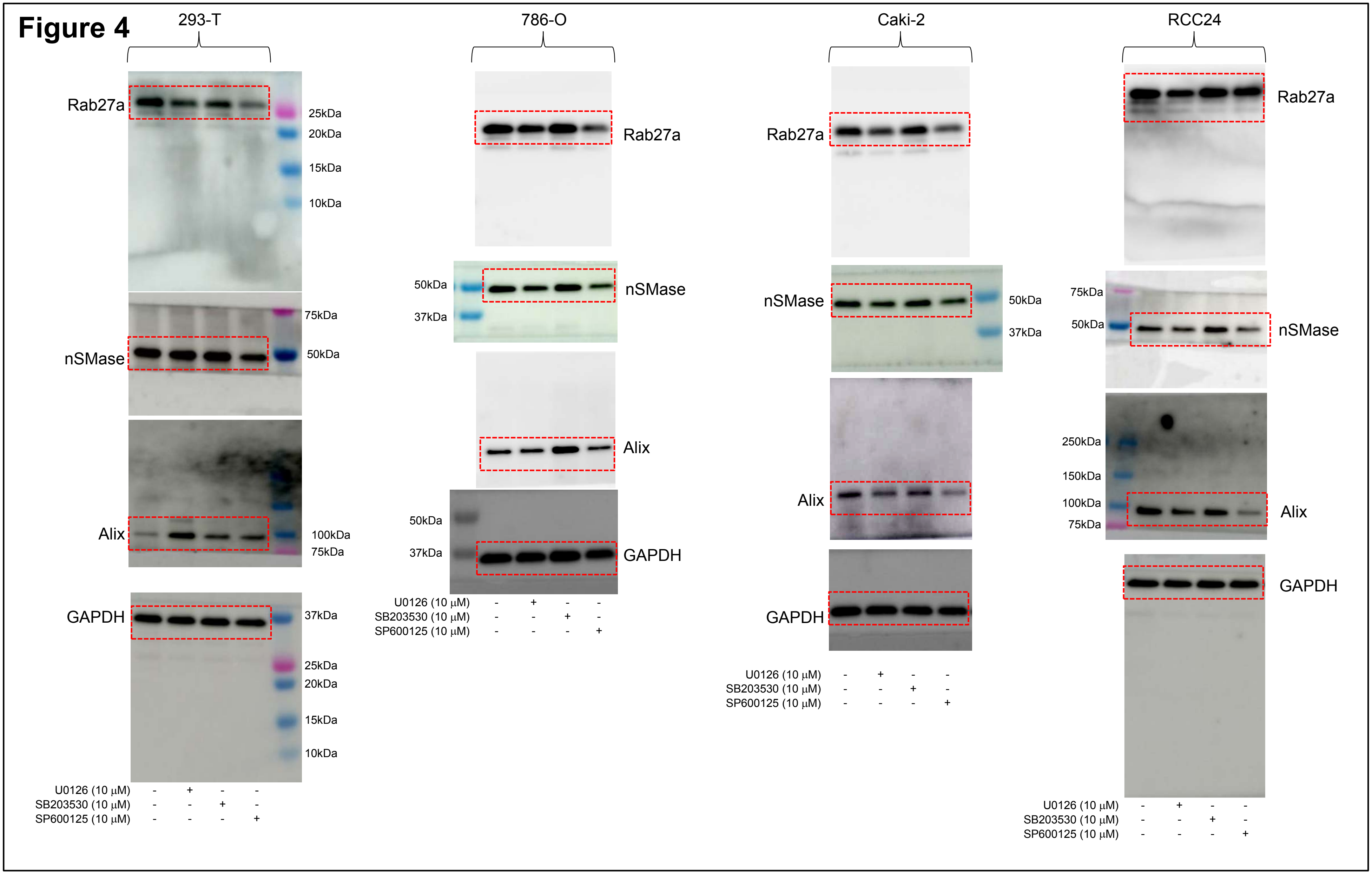
Figure 4
293-T
786-O
Caki-2
RCC24
Rab27a
nSMase
Alix
GAPDH
25kDa
20kDa
15kDa
10kDa
75kDa
50kDa
37kDa
100kDa
250kDa
150kDa
U0126 (10 μM)
SB203530 (10 μM)
SP600125 (10 μM)

Figure S6- 4

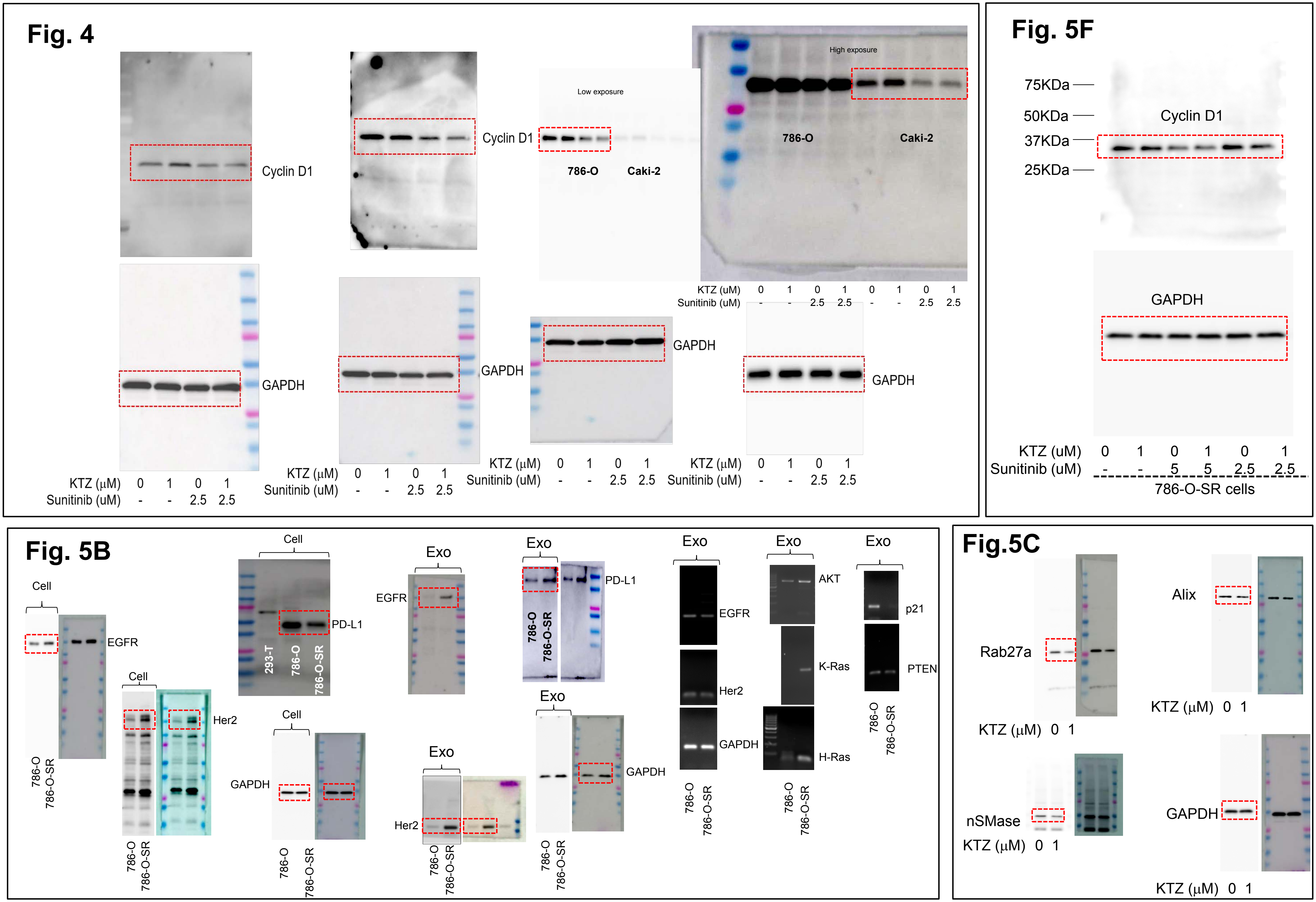

Fig. 4
Cyclin D1
GAPDH
KTZ (μM) 0 1 0 1
Sunitinib (uM) - - 2.5 2.5
Low exposure
High exposure
786-O
Caki-2
KTZ (uM) 0 1 0 1 0 1 0 1
Sunitinib (uM) - - 2.5 2.5 - - 2.5 2.5
Fig. 5F
75KDa
50KDa
37KDa
25KDa
KTZ (uM) 0 1 0 1 0 1
Sunitinib (uM) - - 5 5 2.5 2.5
786-O-SR cells
Fig. 5B
Cell
Exo
EGFR
Her2
PD-L1
GAPDH
AKT
K-Ras
H-Ras
p21
PTEN
293-T
786-O
786-O-SR
Fig.5C
Rab27a
Alix
nSMase
KTZ (μM) 0 1

Table S1

Table. S1 Forward and reverse primer sequences for using PCR

Primer Name		Sequence	Length (bp)
**EGFR**	F	5′-GAGGTGGTCCTTGGGAATTT-3′	125
	R	5′-GGAATTCGCTCCACTGTGTT-3′	
**IGF-1R**	F	5′-AGG AAC AAC GGG GAG AGA GC-3′	97
	R	5′-ACC GGT GCC AGG TTA TGA TG-3′	
**K-ras**	F	5′-GGGGAGGGCTTTCTTTGTGTA-3′	174
	R	5′-GTCCTGAGCCTGTTTTGTGTC-3′	
**H-ras**	F	5′-GGGGCAGTCGCGCCTGTGAA-3′	110
	R	5′-CCGGCGCCCACCACCACCAG-3′	
**AKT**	F	5′-CAACTTCTCTGTGGCGCAGTGC-3′	897
	R	5′-TGGTTGTAGAAGGGCAGGCGAC-3′	
**GAPDH**	F	5′-GACAGTCAGCCGCATCTTCT-3′	127
	R	5′-TTAAAAGCAGCCCTGGTGAC-3′	
**Her2**	F	5’-CCATAACACCCACCTCTGCT-3’	194
	R	5’-ACTGGCTGCAGTTGACACAC-3’	
**PTEN**	F	5′-CAAGATGATGTTTGAAACTATTCCAATG-3′	77
	R	5′-CCTTTAGCTGGCAGACCACAA-3′	
**p21**	F	5’-GGAAGACCATGTGGACCTGT-3’	178
	R	5’-GGATTAGGGCCTCTTGG-3’	
